# Modeling Long-Range Dynamic Correlations of Words in Written Texts with Hawkes Processes

**DOI:** 10.3390/e24070858

**Published:** 2022-06-22

**Authors:** Hiroshi Ogura, Yasutaka Hanada, Hiromi Amano, Masato Kondo

**Affiliations:** Department of Information Science, Faculty of Arts and Sciences, Showa University, Fujiyoshida 403-0005, Japan; hanada@cas.showa-u.ac.jp (Y.H.); kayanm@cas.showa-u.ac.jp (H.A.); mkondo@nr.showa-u.ac.jp (M.K.)

**Keywords:** Hawkes process, autocorrelation function, stochastic process, waiting time distribution, word occurrence, long-range correlation

## Abstract

It has been clarified that words in written texts are classified into two groups called Type-I and Type-II words. The Type-I words are words that exhibit long-range dynamic correlations in written texts while the Type-II words do not show any type of dynamic correlations. Although the stochastic process of yielding Type-II words has been clarified to be a superposition of Poisson point processes with various intensities, there is no definitive model for Type-I words. In this study, we introduce a Hawkes process, which is known as a kind of self-exciting point process, as a candidate for the stochastic process that governs yielding Type-I words; i.e., the purpose of this study is to establish that the Hawkes process is useful to model occurrence patterns of Type-I words in real written texts. The relation between the Hawkes process and an existing model for Type-I words, in which hierarchical structures of written texts are considered to play a central role in yielding dynamic correlations, will also be discussed.

## 1. Introduction

Considering written texts as time series data and analyzing occurrence patterns of any components of texts by using methods of time series analysis have been attempted for various purposes including rhythm analyses [1,2,3,4], analysis of word distributions [5], gathering language statistics for rare words [6], and measuring importance of words [7]. One of the major and actively investigated problems in time series analysis of written texts is to elucidate the origin of long-range dynamic correlations which are observed at various levels of components [8,9,10,11,12,13]. For example, although word-level long-range correlations have been clearly detected especially for words which play important roles in describing the main theme of texts [7,14,15], the stochastic process which brings the dynamic correlations to word occurrences is unknown. More specifically, there exist models of stochastic process yielding long-range dynamic correlations of important words [15,16], but no clear conclusion has yet been obtained. Since occurrences of a word in a written text can be treated as a point process as will be described in the next section, this problem is equivalent to identifying a suitable point process which can reproduce the occurrence patterns of the considered word. We treat this problem in this study, and propose a Hawkes process as a powerful and useful candidate for this point process.

In a wide range of fields, it has gradually been realized that point processes with strong long-range correlations are suitably described by using Hawkes processes [17]. For example, the Hawkes processes have been successfully adopted to model occurrences of earthquakes [18,19,20], neuronal spikes [21,22,23], transactions in financial markets [24], behavior patterns of people on social networking sites [25,26], and patterns of COVID-19 transmission [27]. We expect that the process is also effective for describing word occurrence patterns having dynamic correlations.

Before explaining why this is to be expected, here we briefly describe the definitions of Type-I and Type-II words. Words that have dynamic correlations in their occurrence patterns in a document are called Type-I words, while words that do not exhibit any type of dynamic correlations are called Type-II words. As will be described later, an autocorrelation function (ACF) is used to determine whether a considered word has dynamic correlation or not.

The reasons why we expect the Hawkes process to be applicable to the description of occurrence patterns of Type-I words are as follows.

- Important words which play central roles in describing some notions or ideas in texts are all classified into Type-I words [7,14]. This is because every important word appears in texts with a “burst” nature; once an important word appears in a considered text, then it appears again and again for the duration in which the notion or the idea is described. Thus, the “burst” nature brings dynamic correlations in the occurrence patterns and consequently the word becomes Type-I. The “burst” nature of word occurrences reminds us of the fact that once an earthquake occurs, earthquakes occur more frequently for a short period of time. The Hawkes process can adequately treat such “burst” phenomena because it has a built-in property of self-excitability.
- Type-I words often show long durations of dynamic correlations ranging from several tens to several hundreds of sentences [7,14,15]. These durations correspond to lengths of sentences in which some notion/idea that deeply related with a considered word are described. The Hawkes process is expected to be able to reproduce such long-range dynamic correlations because of its self-excitability.

Note that the Hawkes process is expected to describe only the occurrence patterns of Type-I words, not those of Type-II words. This is because Type-II words appear with almost constant probabilities of occurrence regardless of context, which is different from the self-excited pattern that can be described by the Hawkes process.

In this study, we try to describe occurrence patterns of Type-I words by use of the Hawkes process and check the validity of the description. To our knowledge, this is the first attempt to apply the Hawkes process to analyze written texts. If the description by the Hawkes process is successful, it will not only be an important application of the Hawkes process, but also be an important step in attempts to describe document generation by stochastic models.

The rest of the paper is organized as follows. In the next section, we present the method of how to find/optimize an adequate Hawkes process for a considered Type-I word, given occurrence patterns of the word. The section also presents procedures that check the validity of the optimized Hawkes process through simulation. Section 3 is devoted to describing the results of validation of the optimized Hawkes processes. In Section 4, we discuss the relation between the Hawkes process and an existing stochastic model of generating Type-I words in which a hierarchical structure of written texts (volumes, chapters, sections, subsections, paragraphs, sentences) is taken into account. In the last section, we give our conclusion and indicate a direction of future study.

## 2. Methodology

The main purpose of this study is to verify whether the process of generating Type-I words can be regarded as a Hawkes process or not. To achieve the purpose, we take the following 3 steps.

1. From the occurrence patterns of Type-I words in real written texts, we calculate autocorrelation functions (ACFs) to characterize dynamic correlations of these words. Details are given in Section 2.1.
2. We optimize a Hawkes process so that it can express the stochastic process of yielding observed occurrence patterns of a considered word. For the optimization, we utilize a log-likelihood function of the Hawkes process, and maximize the function by optimizing parameters of the process, given the observed word occurrence signals. Then, the Hawkes process, having the kernel function with the optimized values of parameters, is considered to be the best way to express the observed word occurrence signal of the considered word in the sense of maximum likelihood estimation (MLE). A detailed description is given in Section 2.2.
3. We generate word occurrence signals of the considered word from the optimized Hawkes process. This is achieved by standard simulation procedures of point processes [28]. The simulated word occurrence signals are then used to calculate ACFs. The ACFs of the simulated signals are compared with the ACFs obtained in step 1 to validate the optimized Hawkes process. Section 2.3 explains the procedure in detail.

As described above, we mainly utilize ACF as a characteristic quantity of a stochastic process. In general, waiting time distributions (WTDs) are also used along with ACFs in time series analysis because they both contain equivalent information in principle. However, ACFs is more suitable in this study, because ACFs offer more precise descriptions of dynamic correlations for the case of word occurrence signals [16].

### 2.1. Model Functions of ACFs for Type-I and Type-II Words

One of the methods to convert occurrence patterns of a considered word in the considered text to time series data is to utilize the following definition of a binary time-dependent signal X(t):

(1) $$X\left(t\right)=\{\begin{array}{c} 0\;\;\;\left(\mathrm{if}\;a\;\mathrm{considered}\;\mathrm{word}\;\mathrm{does}\;\mathrm{not}\;\mathrm{occur}\;\mathrm{in}\;\mathrm{the}\;t\mathrm{th}\;\mathrm{sentence}\right)\\ 1\;\;\;\;\;\;\;\;\;\;\;\;\;\;\;\;\;\;\;\left(\mathrm{if}\;a\;\mathrm{considered}\;\mathrm{word}\;\mathrm{occurs}\;\mathrm{in}\;\mathrm{the}\;t\mathrm{th}\;\mathrm{sentence}\right) \end{array}.$$

Here, t is an ordinal number of sentences that assigned from the first to the last sentences in a considered text and it plays a role of time along the text. By defining a binary time-dependent signal X(t) as in Equation (1), we can utilize various results in point process theory for our investigation.

**Figure 1:**
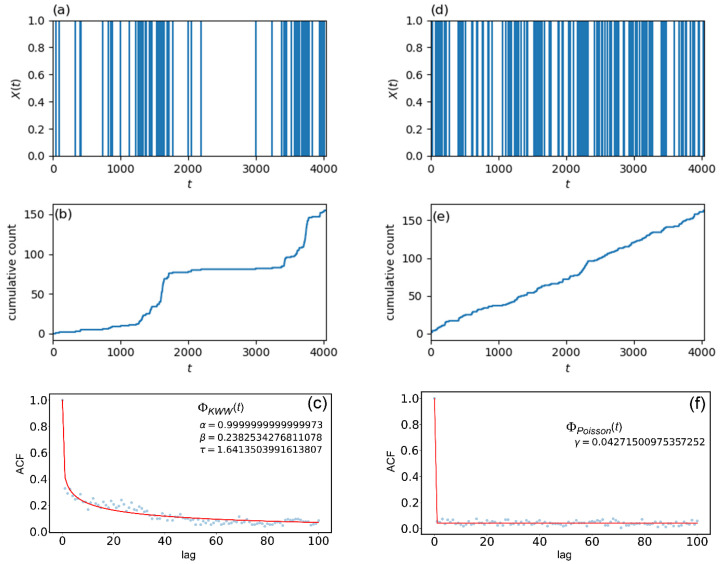
Word occurrence signals X(t) (**a**,**d**), as defined by Equation (1); cumulative count of word occurrences (**b**,**e**), and ACFs (**c**,**f**) of the words “organ” (**a**–**c**) and “seem” (**d**–**f**). The words “organ” and “seem” are typical Type-I and Type-II words, respectively, picked from the Darwin text. Occurrences of “organ” are in a context-specific and bursty manner (**a**,**b**), and long-range dynamic correlations are seen in the ACF plot (**c**), while occurrences of “seem” show an approximately constant occurrence rate (**d**,**e**) and no type of dynamic correlation is seen in ACF (**f**). Red lines in (**c**,**f**) show best fitted results by use of Equations (3) and (4), respectively.

Two examples of word occurrence signals X(t) are shown in Figure 1a,d. The two words, “organ” and “seem” used in the figure, are both picked from “On the Origin of Species” by Charles Darwin. The word “organ” is a typical Type-I word in the book, and thus it shows a “burst” nature in X(t) (Figure 1a) and in a cumulative count of the word occurrences along the text (Figure 1b). On the other hand, since the word “seem” is a typical Type-II word in the book, its X(t) (Figure 1d) and cumulative count (Figure 1e) do not exhibit a “bursty” nature but show word occurrences with a constant occurrence rate, which indicates the occurrences are purely governed by chance.

Figure 1c,f shows ACFs of word “organ” and “seem”, which are calculated from X(t) displayed in Figure 1a,d, respectively. Since X(t) is a discrete-time signal, a general definition of ACF for a continuous-time signal A(t) given by

(2) $$\Phi \left(t\right)=\frac{\underset{T\to \infty }{\lim}\int_{0}^{T}A\left(\tau \right)A\left(\tau +t\right)d\tau }{\underset{T\to \infty }{\lim}\int_{0}^{T}A\left(\tau \right)A\left(\tau \right)d\tau },$$

is extended for our discrete-time case and the extended definition is used for the calculations [7]. The ACF shown in Figure 1c indicates that the dynamic correlation gradually decreases as lag increases, which is a typical behavior of ACFs in usual linear systems. On the other hand, ACF in Figure 1f shows abrupt decrease from its initial value one at t=0 to some constant value of almost zero at t>0. This behavior of ACF indicates that Type-II words are generated from stochastic processes that do not have any type of dynamic correlations.

To represent characteristics of ACFs of Type-I words, we introduce an empirical model function which gives satisfactory fittings of observed ACFs for Type-I words [7,14]. The function is called the Kohlrausch–Williams–Watts (KWW) function and has a stretched exponential form given by

(3) $$\;\Phi_{KWW}\left(t\right)=\alpha \exp\left\{-{\left(\frac{t}{\tau }\right)}^{\beta }\right\}+\left(1-\alpha \right),$$

where α,β and τ are fitting parameters satisfying 0<α≤1, 0<β≤1 and 0<τ. The fitting result for the Type-I word “organ” by use of Equation (3) is also shown as a red curve in Figure 1c with optimized values of fitting parameters.

A model function for ACFs of Type-II words is given by

(4) $$\Phi_{Poisson}\left(t\right)=\{\begin{array}{c} 1\;\;\;\left(t=0\right)\\ \gamma \;\;\;\left(t>0\right) \end{array},$$

where γ is a fitting parameter satisfying 0<γ<1 and it actually takes a value almost equal to zero. Note that Equation (4) is theoretically derived under an assumption that the stochastic process yielding Type-II words is a Poisson point process [7]. In Figure 1f, the result of fitting with Equation (4) is displayed as a red line.

Classifying whether a given word is Type-I or Type-II is performed as follows. First, we execute two non-linear least squares fittings by use of Equations (3) and (4) simultaneously on the one observed ACF of a considered word and compare two values of the Bayesian information criterion (BIC) each of which obtained at the fitting using Equation (3) and at the fitting using Equation (4). If the BIC of the fitting using Equation (3) is smaller than that using Equation (4), then the word is classified as Type-I and otherwise the word is classified as Type-II. We can classify an arbitrary word in written texts as Type-I or Type-II without any ambiguities by using this procedure with some additional criteria [7].

Seven texts employed in this study, which are famous academic books chosen so as to represent wide range of written texts, are listed in Table 1 with their short names and some information. The procedure for preparing texts is the same as in the previous paper [16]. In this study, we determined Type-I words to be analyzed through following steps. First, words that appear at least 50 sentences or more in each of the 7 texts are chosen as frequent words. All frequent words are then classified whether Type-I or Type-II by the classification method described above. Finally, stop words are removed from the set of the Type-I words, and remaining words are used for further analysis. As stop words, we used the same list of stop words that the MySQL 8.0 system uses for full-text queries (https://dev.mysql.com/doc/refman/8.0/en/fulltext-stopwords.html accessed on 2 March 2022).

### 2.2. Log-Likelihood Function of Hawkes Process

**Figure 2:**
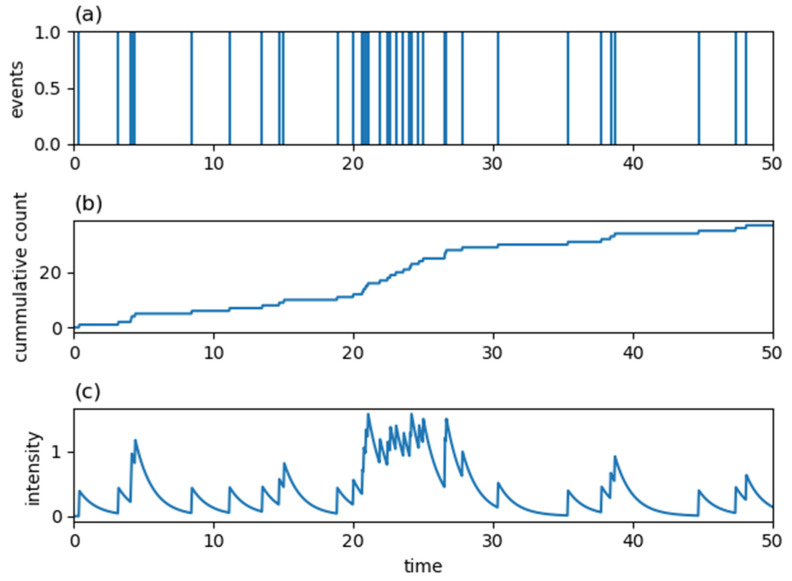
Examples of (**a**) the occurrence signal of events, (**b**) the cumulative count of events and (**c**) the conditional intensity function for the Hawkes process with $\lambda \left(t|H_{t}\right)=0.2+\underset{t_{i}<t}{\sum }0.4\;\exp\left\{-0.8\left(t-t_{i}\right)\right\}$.

**Figure 3:**
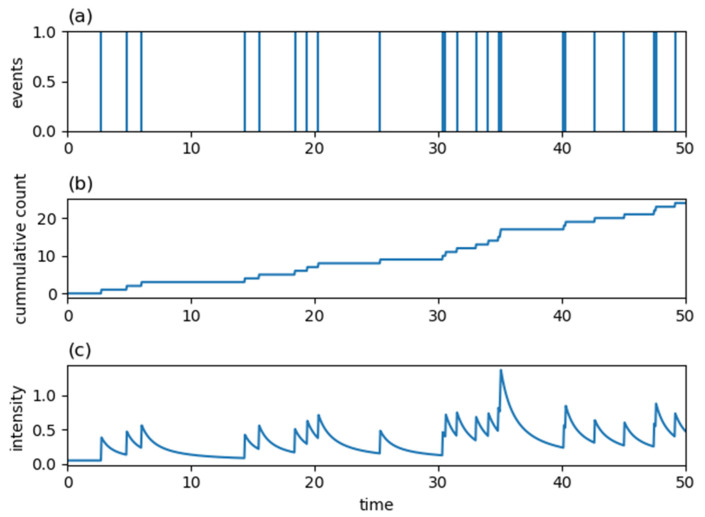
Examples of (**a**) the occurrence signal of events, (**b**) the cumulative count of events and (**c**) the conditional intensity function for the Hawkes process with $\lambda \left(t|H_{t}\right)=0.05+\underset{t_{i}<t}{\sum }\frac{0.9}{{\left(t-t_{i}+1.7\right)}^{1.8}}$.

Here we recall the basic notation of Hawkes process for later reference [25,29]. A Hawkes process is a kind of self-exciting point process and has been applied in diverse areas because of its self-exciting nature. This ‘self-excite’ means that each arrival/occurrence increases the rate of future arrivals/occurrences for some period. The conditional intensity function of the Hawkes process is given by

(5) $$\lambda \left(t|H_{t}\right)=\mu +\underset{t_{i}<t}{\sum }g\left(t-t_{i}\right),$$

where $H_{t}$ denotes the history of all events occurring before time t, μ is the background intensity,$\;t_{i}$ means a time of the ith event occurrence before time t, and g(τ) is a kernel function which determines how past events will affect the future. Two frequent choices of the kernel function, which are also used in this study, are

(6) $$g_{exp}\left(\tau \right)=ab\exp\left(-b\tau \right),\;$$

(7) $$g_{pow}\left(\tau \right)=\frac{K}{{\left(\tau +c\right)}^{p}},\;$$

where a and b in Equation (6) and c,p and K in Equation (7) are parameters of the kernels taking non-negative real values. Examples of an occurrence signal, a cumulative count, and a conditional intensity function for a Hawkes process with an exponentially decaying kernel, Equation (6), are shown in Figure 2. Figure 3 shows corresponding quantities to Figure 2 for a Hawkes process with a power-law decaying kernel, Equation (7). As seen in these figures, since a value of the intensity function at the current time is enhanced by the history of past generated events, once an event is generated, it tends to be generated intensively in a short period of time in Hawkes processes.

As mentioned before, the main objective of this study is to verify whether the Hawkes process is eligible to describe stochastic processes yielding Type-I words or not. The first step of the verification is to find the Hawkes process that best approximates the stochastic process yielding the actual occurrence signal of a considered word within the descriptive power of Hawkes processes. The most suitable Hawkes process, which is expected to be able to reproduce real word occurrence signals X(t) of a considered Type-I word, is searched for by maximizing the log-likelihood function of the Hawkes process. Therefore, a searching method to find the optimized Hawkes process described below is in the manner of standard maximum likelihood estimation (MLE).

Given the history of all events in the time interval [0,T], i.e., given the record of all occurrence times

(8) $$D^{\left[0,T\right]}={\left\{t_{i}\right\}}_{i=1}^{n},$$

the log-likelihood function of the Hawkes process having conditional intensity function of Equation (5) is given by [30]

(9) $$l\left(\theta |D^{\left[0,T\right]}\right)=\log L(\theta |D^{\left[0,T\right]})=\overset{n}{\underset{i=1}{\sum }}\log\lambda (t_{i}|H_{t_{i}})-\overset{T}{\underset{0}{\int }}\lambda \left(t|H_{t}\right)dt,$$

where θ denotes a set of parameters of the Hawkes process. If we combine Equations (5) and (6), then Equation (9) becomes

(10) $$\begin{array}{ll} l_{exp}\left(\mu ,\;a,b|{\left\{t_{i}\right\}}_{i=1}^{n}\right) & \\ & =\sum_{i=1}^{n}\log\left[\mu +\sum_{j<i}ab\exp\left\{-b\left(t_{i}-t_{j}\right)\right\}\right]\\ & -\left[\mu T+\sum_{i=1}^{n}a\left\{1-\exp\left(-b\left(T-t_{i}\right)\right)\right\}\right]. \end{array}$$

In the same way, combining Equations (5) and (7) makes Equation (9) to

(11) $$\begin{array}{ll} l_{pow}\left(\mu ,c,K,p|{\left\{t_{i}\right\}}_{i=1}^{n}\right) & \\ & =\sum_{i=1}^{n}\log\left[\mu +\sum_{j<i}\frac{K}{{\left(t_{i}-t_{j}+c\right)}^{p}}\right]\\ & -\left[\mu T+\sum_{i=1}^{n}\frac{K}{p-1}\left\{\frac{1}{c^{p-1}}-\frac{1}{{\left(T-t_{i}+c\right)}^{p-1}}\right\}\right]. \end{array}$$

In our case, the set of occurrence times of events, ${\left\{t_{i}\right\}}_{i=1}^{n}$ in Equations (10) and (11), is equivalent to the set of times (sentence numbers) at which word occurrence signal X(t) takes value one. MLE of $\theta_{exp}=\left(\mu ,a,b\right)$ for exponentially decaying kernel, Equation (6), and that of $\theta_{pow}=\left(\mu ,c,K,p\right)$ for power-low decaying kernel, Equation (7), are thus obtained by substituting ${\left\{t_{i}\right\}}_{i=1}^{n}$ to Equations (10) and (11), respectively, and then maximizing these functions. To maximize Equation (10) as a function of $\theta_{exp}=\left(\mu ,a,b\right)$, and to maximize Equation (11) as a function of $\theta_{pow}=\left(\mu ,c,K,p\right)$, we use a quasi-Newton method with the BFGS algorithm [31].

In actual procedures of the quasi-Newton method, we introduce new parameters $m_{0},\;a_{0},\;b_{0},\;c_{0},\;K_{0}$ and $p_{0}$ instead of using original parameters μ,a,b,c,K and p in order to stabilize convergence calculations. The original and new parameters are related as follows.

(12) $$\mu =0.5+0.5\tanh(m_{0}),$$

(13) $$a=0.5+0.5\tanh(a_{0}),$$

(14) $$b=\exp\left(b_{0}\right),$$

(15) $$c=\exp\left(c_{0}\right),$$

(16) $$K=0.5+0.5\tanh(K_{0}),$$

(17) $$p=3.2+2.0\tanh(p_{0}).$$

Note that all of the original parameters have a restriction that they should be non-negative, but newly introduced parameters can take any real values. Equations (12)–(17) also indicate that the conditions of the original parameters expressed by the following inequalities are automatically satisfied; 0<μ<1, 0<a<1, 0<b, 0<c, 0<K<1, 1.2<p<5.2. The last condition for p, 1.2<p<5.2, is needed in a practical sense because the Hawkes process becomes unstable when p<1. To determine which of the two kernel functions, Equation (6) and Equation (7), is more appropriate given actual word occurrence data ${\left\{t_{i}\right\}}_{i=1}^{n}$, we use the Akaike’s Information Criterion (AIC) for the judgement; i.e., we select Equation (6) as a better kernel function when the AIC of MLE calculation with Equation (10) is smaller than that with Equation (11), and otherwise we select Equation (7). The AIC is an estimator of prediction error and therefore it provides a mean for model selection in the same way as BIC [32]. The reason for using AIC instead of using BIC is that AIC has a proven track record of being applied in model selection involving Hawkes processes [30].

### 2.3. Simulating Word Occurrence Events from Hawkes Process

The conditional intensity function, Equation (5), can be determined for each of the Type-I words by using optimized kernel parameters $\theta_{exp}=\left(\mu ,a,b\right)$ in Equation (6) or $\;\theta_{pow}=\left(\mu ,c,K,p\right)$ in Equation (7), which are obtained by MLE procedures with the word occurrence signal ${\left\{t_{i}\right\}}_{i=1}^{n}$ of a considered word. Once the conditional intensity function, Equation (5), is fixed, then we can simulate word occurrence signal X(t) from the Hawkes process having that fixed intensity function. The simulation period T was set to be equal to the actual text length (length in sentences of a considered book) to check the validity of the Hawkes process; i.e., if the Hawkes process employed in the simulation is valid, then the number of occurrences in simulated X(t) is almost equal to the number of occurrences of a considered word in real written text. For simulating X(t), we use a standard thinning algorithm for simulation of point processes [28]. Since the simulated word occurrence times $t_{i}$ can take any real values between 0 and T, we convert these values of $t_{i}$ to the nearest integer values in order to meet the condition that real $t_{i}$ take only integer values in time along the text.

Then, we calculate ACFs from the simulated signal X(t) and we further perform the curve fitting with Equation (3), as described in Section 2.1. We then evaluate the degree of agreement between the ACFs calculated from actual word occurrence signals X(t) and the ACFs calculated from simulated X(t) from the Hawkes process. The evaluation is made through comparisons of two sets of optimized fitting parameters; one is the set of parameters in Equation (3) obtained for real ACFs and the other is the set of parameters obtained for simulated ACFs calculated from simulated X(t).

## 3. Results

### 3.1. MLE of Hawkes Process

**Figure 4:**
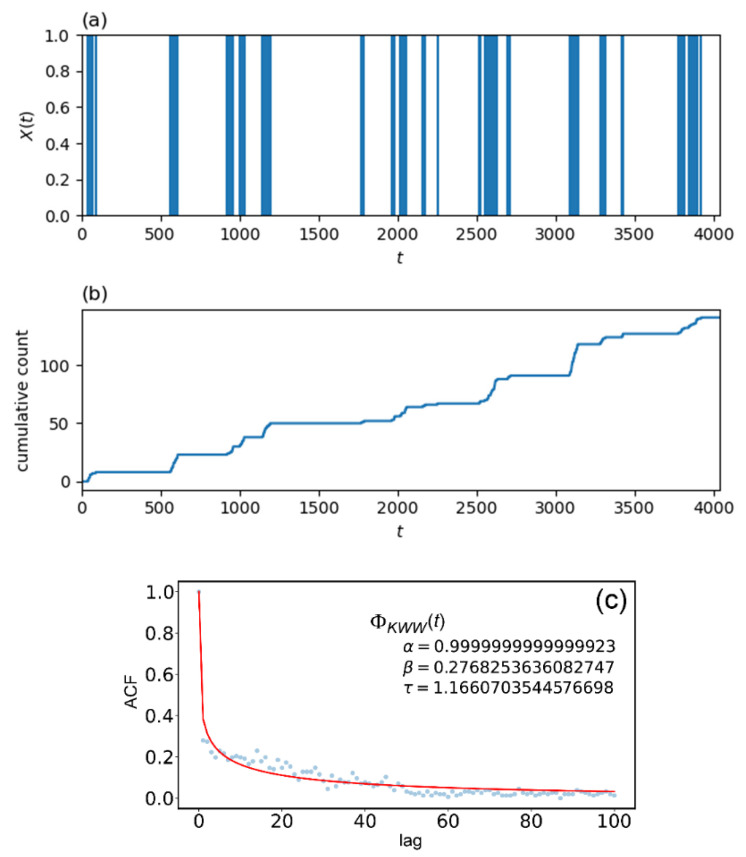
Results of the simulation for generating word occurrence signal of “organ” in Darwin text. (**a**) Simulated word occurrence signal X(t) obtained from the optimized Hawkes process for “organ”, (**b**) cumulative count of word occurrences obtained from simulated X(t), and (**c**) ACFs calculated from simulated X(t) and the best fitted curve by use of Equation (3).

Table 2 summarizes the results of MLE performed given the real word occurrence signal X(t) of the word “organ” which is shown in Figure 1a. The parameter values listed in the table are optimized ones to maximize Equations (10) and (11). Comparing the two AIC values in the table, we can judge that $g_{exp}\left(\tau \right)$ is more suitable than $g_{pow}\left(\tau \right)$ to simulate the real word occurrence signal X(t). Thus, we employ the Hawkes process with $g_{exp}\left(\tau \right)$ having the parameter values of (μ,a,b) shown in Table 2 to simulate X(t). The simulated X(t), cumulative count obtained from the simulated X(t) and ACF calculated from the simulated X(t) are shown in Figure 4a–c, respectively. Figure 4c also shows the results of the curve fitting using Equation (3). Although the “burst” periods are different in Figure 1 and Figure 4, overall behaviors of ACFs are almost coincide in Figure 1c and Figure 4c.

Table 3 shows comparison of the curve fitting results of two different ACFs; one is the ACFs calculated from the real X(t) of the word “organ”, and the other is the ACF obtained from simulated X(t)  for the same word. From Table 3, it can be seen that the number of word occurrences are almost the same for real and simulated X(t), and the values of other parameters are also similar. This result indicates that the Hawkes process with optimized parameters can reproduce a word occurrence signal having the same statistical properties as the actual signal of the word “organ”.

### 3.2. Results of Simulations for All Type-I Words

**Figure 5:**
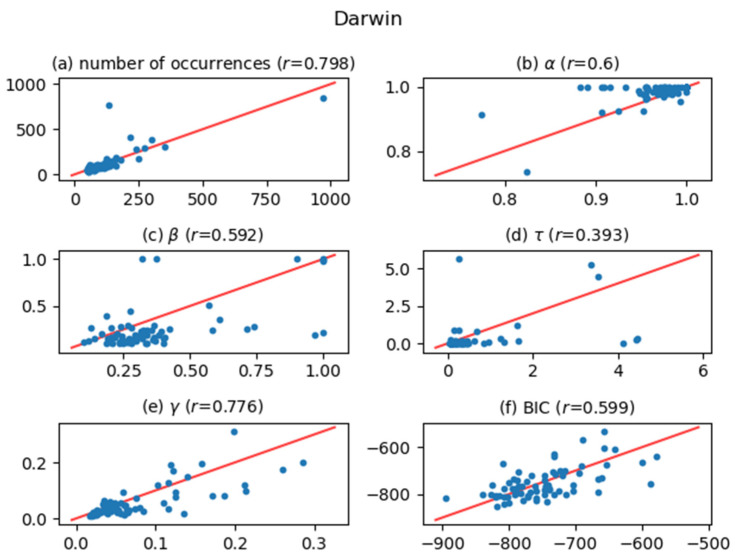
Comparisons between values evaluated from real X(t) (horizontal) and those evaluated from simulated X(t) (vertical) across 6 characteristic quantities. All quantities were calculated for each of Type-I words that appears in Darwin text. The red line in each plot represents the relation y=x. The title of each plot includes a value of correlation coefficient, r, which indicates strong positive correlation when r is larger than about 0.6.

**Figure 6:**
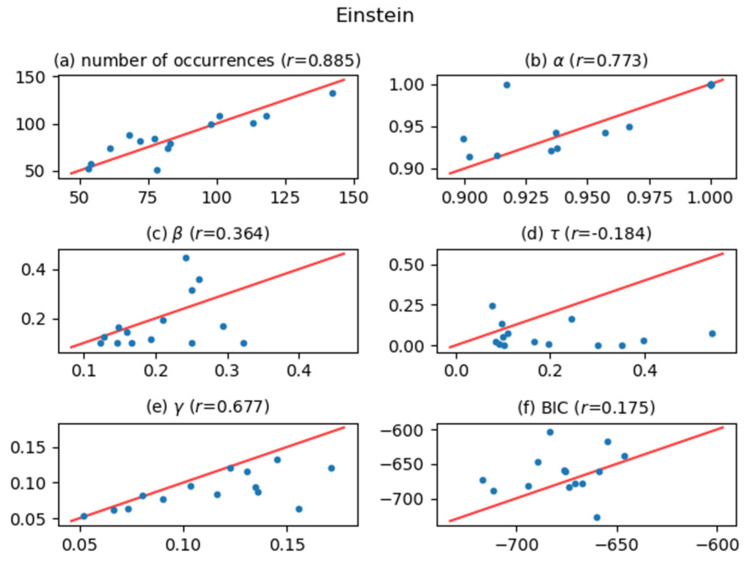
The same meaning plots as in Figure 5 for the case of the Einstein text.

**Figure 7:**
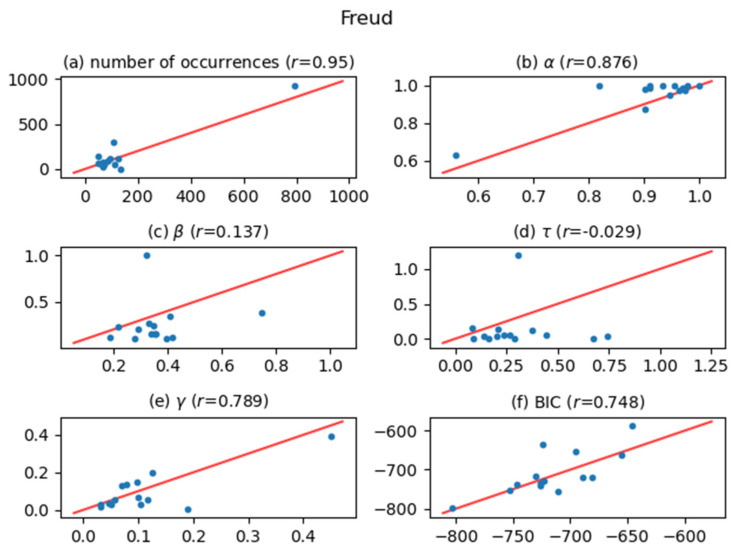
The same meaning plots as in Figure 5 for the case of the Freud text.

**Figure 8:**
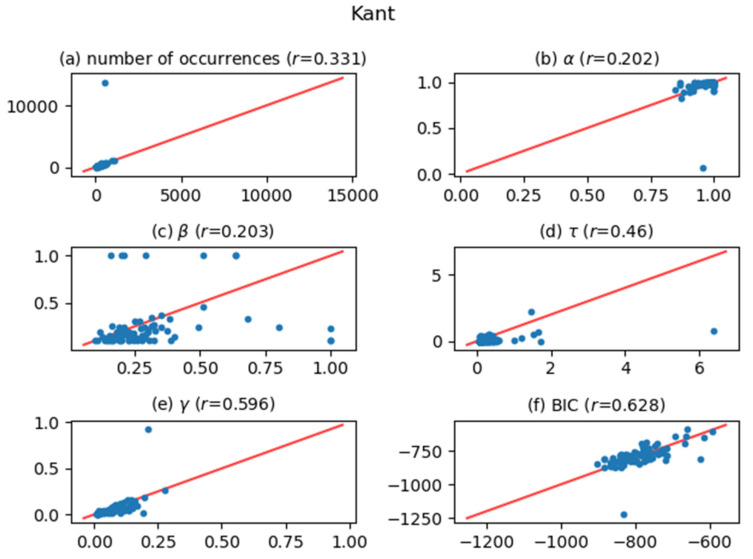
The same meaning plots as in Figure 5 for the case of the Kant text.

**Figure 9:**
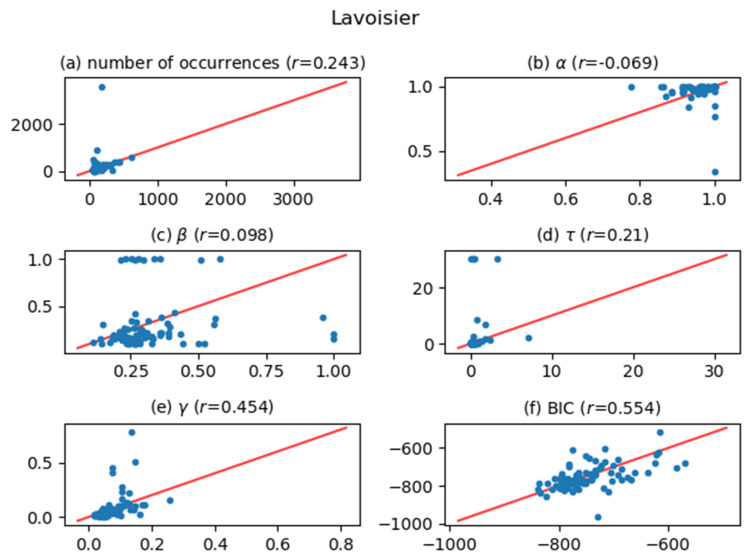
The same meaning plots as in Figure 5 for the case of the Lavoisier text.

**Figure 10:**
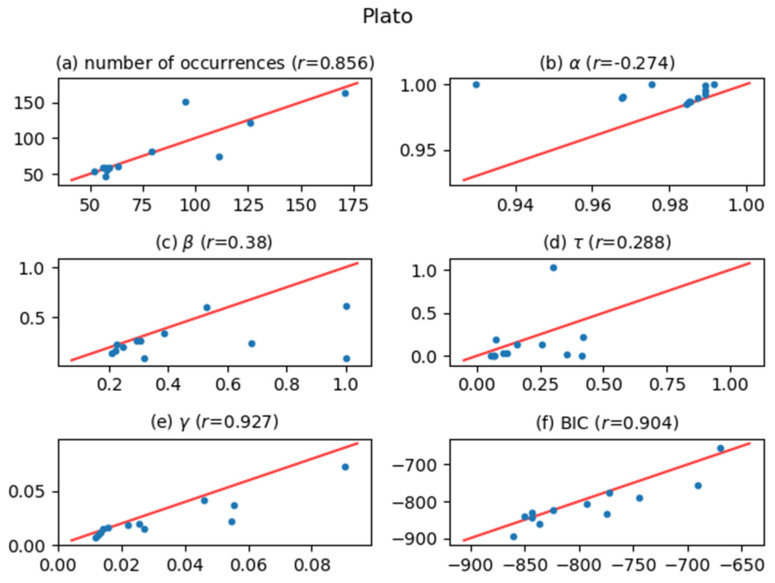
The same meaning plots as in Figure 5 for the case of the Plato text.

**Figure 11:**
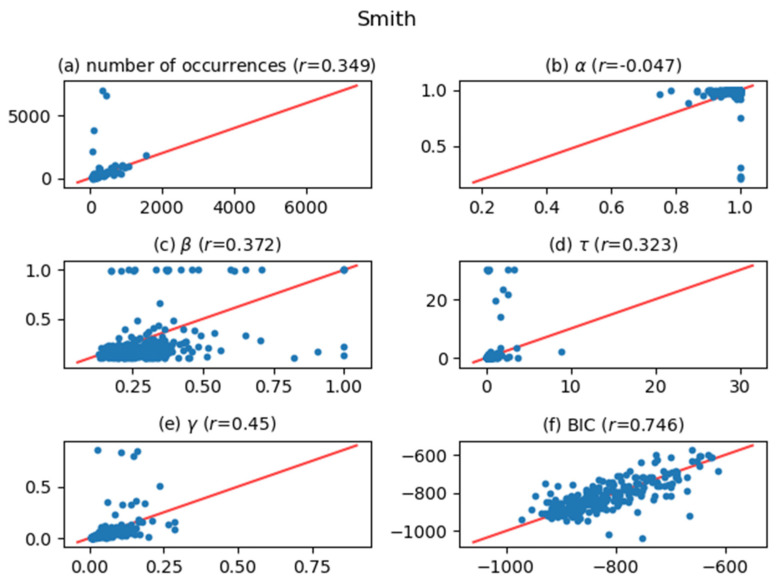
The same meaning plots as in Figure 5 for the case of the Smith text.

Figure 5, Figure 6, Figure 7, Figure 8, Figure 9, Figure 10 and Figure 11 show comparisons of the curve fitting results of two different ACFs for all Type-I words which appear in 7 texts listed in Table 1. In each plot of (a) in these figures, the horizontal axis represents occurrence numbers of words in real written text, while the vertical axis represents occurrence numbers in the simulated X(t). In each plot of (b), (c), and (d), the horizontal axis represents one of the fitting parameters of ACFs obtained from the real signal X(t), while the vertical axis represents that of ACFs obtained from simulated X(t). In each plot of (e), the horizontal axis shows values of fitting parameter γ, which appears in Equation (4), for ACFs obtained from the real signal X(t), while the vertical axis shows values of γ  for ACFs obtained from simulated X(t). One may wonder why we use here the parameter γ which appears in the model function for Type-II words, Equation (4). The value of γ is equal to the intensity rate λ in a simple Poisson point process [7] and can therefore be regarded as the averaged value of the conditional intensity function $\lambda \left(t|H_{t}\right)$ of a Hawkes process. Thus, if the horizontal and vertical values in plot (e) are approximately equal, then overall behaviors of real and simulated X(t) are considered to be similar. In each plot of (f), the horizontal axis shows BICs of fitting ACFs obtained from the real signal X(t), while the vertical axis shows BICs of fitting ACFs obtained from simulated X(t). If the horizontal and vertical values in plot (f) are approximately equal, then overall behaviors of ACFs obtained from real and simulated X(t) are considered to be similar. In all plots, one plot point corresponds to one Type-I word; for example, horizontal and vertical values of one plot point in a plot (a) represent the real occurrence number of a considered Type-I word (the horizontal value) and occurrence number in the simulated signal for that word (the vertical value). The red line in each plot shows the relation y=x, on which plot points should be located when Hawkes processes are sufficient to represent original word yielding processes. The correlation coefficients for the vertical and horizontal quantities are also displayed in the title of each plot.

The degree of agreement between the vertical and horizontal quantities of each plot in Figure 5, Figure 6, Figure 7, Figure 8, Figure 9, Figure 10 and Figure 11 indicates that the actual signals of Type-I words can be reproduced somewhat accurately using the optimized Hawkes processes. In particular, for the reasons listed below, we can conclude that the Hawkes processes have sufficient descriptive power to express original stochastic processes yielding Type-I words.

- The correlation coefficients of the number of occurrences (plot (a)) show strong positive correlations in most texts. This fact indicates that actual and simulated X(t) share same statistical properties.
- The correlation coefficients of γ (plot (e)) and those of BIC (plot (f)) also show strong positive correlations. This indicates that ACFs of real X(t) and those of simulated X(t)  are very similar in overall behaviors.

The most significant reason why the horizontal and vertical quantities do not perfectly match in these figures is that the simulated X(t) generated by a Hawkes process with optimized parameters is only one sample of the time series data among infinite realizations generated by the optimized Hawkes process. If we prepare sufficient samples, i.e., large number of simulated signals X(t) from the optimized Hawkes process for one Type-I word, and use averaged values for all simulated X(t) to obtain one vertical value, then the vertical value tends to approach to the corresponding horizontal value of the considered word. This means that each of all runs generating X(t) offers one vertical value, and averaging all vertical values justifies their convergence toward the real value. However, this requires high computational costs, and is out of the scope of this study.

## 4. Discussion

In our previous study [16], a model of stochastic process that yields Type-I words has been proposed. The characteristic of the model is that a waiting time distribution (WTD) of word occurrences has a fractal structure, which is naturally introduced from the hierarchical structure of written texts, i.e., volumes, chapters, sections, subsections, paragraphs, and sentences. More specifically, the fractal nature of WTDs has been clarified through the following procedures [16].

1. First, we construct an intensity function of word occurrence along a text, P(t), which describes the occurrence probability of a considered word at time t. The construction is done in a recursive way in which the hierarchical structure of written texts is considered. Note that P(t) represents word occurrence probability per unit time at time t, and thus it corresponds to $\lambda (t|H_{t})$ of Hawkes processes.
2. A Monte Carlo simulation is performed by use of P(t) to generate a word occurrence signal X(t).
3. Waiting times $t_{w}$, which denote a time between two successive word occurrences, and their distribution $P\left(t_{w}\right)$ are calculated from the X(t).
4. The resultant log-log plot of $t_{w}$ vs. $P\left(t_{w}\right)$ shows a linear relationship, indicating that the WTD has a fractal structure.

**Figure 12:**
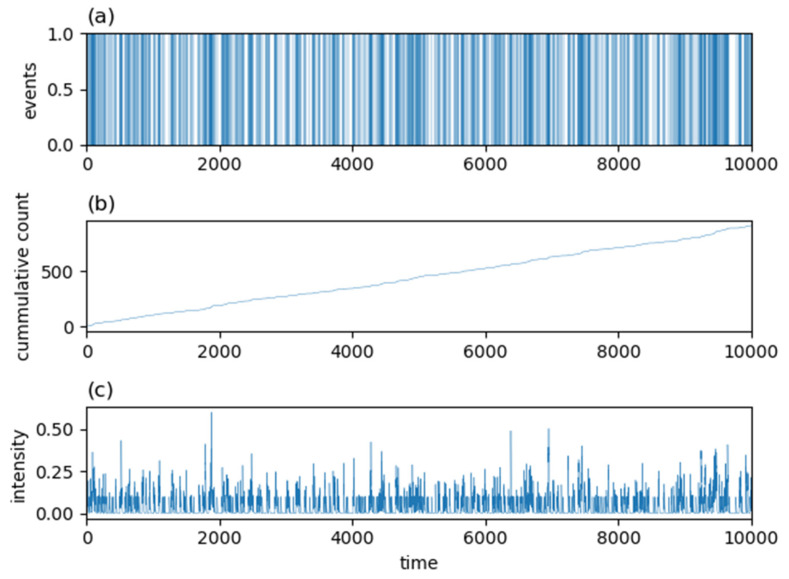
Simulated signal X(t) (**a**), cumulative count of word occurrences (**b**), and conditional intensity function $\lambda \left(t|H_{t}\right)$ (**c**), of the Hawkes process defined by $\lambda \left(t|H_{t}\right)=0.05+\underset{t_{i}<t}{\sum }0.1\;\exp\left\{-0.25\left(t-t_{i}\right)\right\}$.

Since the Hawkes process is defined by Equations (5)–(7), and since the structure of written texts is not taken into account among these equations, the above model does not seem to be related to the Hawkes process. However, the conditional intensity function $\lambda (t|H_{t})$ of Hawkes processes employed in this study and P(t) described in the first procedure above, are very similar to each other. To illustrate this fact, and to follow procedures 1 to 4 mentioned above by use of simulated signals from the Hawkes process, we present another simulation result in which the simulated period is set to be a longer value of T=10,000  to make result clearer. Figure 12 shows the simulated signal X(t) from a Hawkes process, cumulative count of word occurrences, and $\lambda (t|H_{t})$ of the process over the period of [0, 10,000]. The conditional intensity function $\lambda (t|H_{t})$ shown in Figure 12c is very similar to the previously reported P(t) [14,16] in the point that it seems to be restricted to take several approximately discretized values.

**Figure 13:**
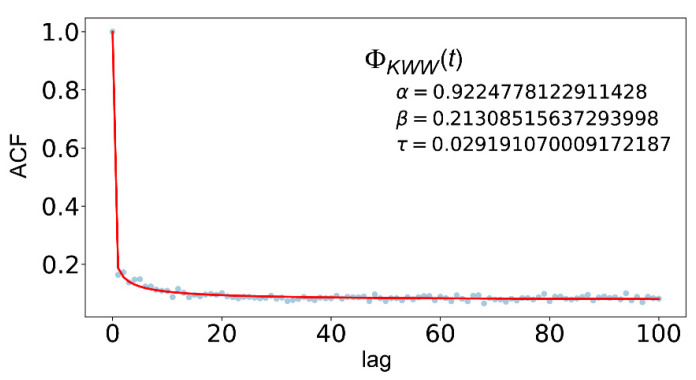
ACF calculated from X(t) shown in Figure 12a and its fitting result with Equation (3).

Figure 13 shows ACF calculated from simulated X(t) shown in Figure 12a and its fitting results by use of Equation (3). Note that the fitting parameter τ takes a very small value of about τ≅0.03 in Figure 13. In general, when τ in Equation (3) becomes smaller, then the resultant $\Phi_{KWW}\left(t\right)$ defined by Equation (3) approaches to $\Phi_{Poisson}\left(t\right)$ given by Equation (4), and at the limit of τ→0, Equation (3) becomes Equation (4). Thus, the ACF shown in Figure 13 has intermediate properties between Figure 1c,f. In the same context, Figure 12a has intermediate properties between Figure 1a,d, and Figure 12b is in between Figure 1b,e. Therefore, the simulated signal shown in Figure 12a has a dynamic correlation with “intermediate” strength.

**Figure 14:**
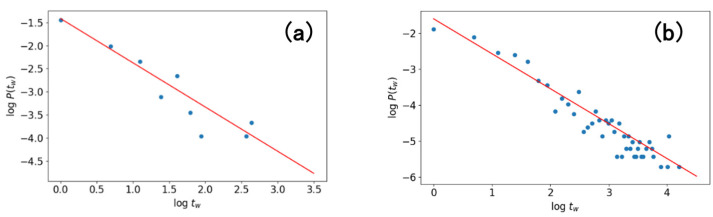
Double logarithmic plot of waiting time $t_{w}$ and its distribution $P\left(t_{w}\right)$ which are calculated from simulated X(t). The plot (a) uses X(t) shown in Figure 4a and the plot (**b**) uses X(t) shown in Figure 12a. Note that sample size is smaller in (**a**) than in (**b**) because the length of the used text is also shorter for (**a**) (l=4036 ) than for (**b**) (l=10,000). The red lines shown in the figure indicate the relations of $\log P\left(t_{w}\right)=-0.95661\log t_{w}-1.41603$ in plot (**a**) and $\log P\left(t_{w}\right)=-0.97394\log t_{w}-1.60345$ in plot (**b**) which were obtained by the method of weighted least square. To avoid noise affecting the fittings, we omit $t_{w}$ having an occurrence count less than or equal to 2.

Figure 14 shows two examples of the relationship between waiting time $t_{w}$ versus their distribution $P\left(t_{w}\right)$ in double logarithmic plots. The values of $t_{w}$ and $P\left(t_{w}\right)$ used in the figure are obtained from two simulated X(t) from Hawkes processes; one is the X(t) simulated for the word “organ” (Figure 4a) and the other is X(t) shown in Figure 12a. Note that a linear relationship seems to hold between $\log t_{w}\;$ and $\log P\left(t_{w}\right)$ for both cases, indicating that a fractal structure exists in the WTDs. It is confirmed that, therefore, the Hawkes processes bring some fractal structure in WTDs even in the case at which the Hawkes process does not have strong dynamic correlations but an “intermediate” correlation level. From the results shown in Figure 14, it seems almost certain that the Hawkes processes optimized to describe the generations of Type-I words have some fractal structures within their WTDs. However, in order to clearly demonstrate this conclusion, large-scale simulations with longer periods T are needed.

## 5. Conclusions

Occurrence patterns of Type-I words that appear in seven famous academic books were simulated by use of Hawkes processes. To seek an optimized Hawkes process for each of the Type-I words, we performed maximum likelihood estimation of the parameters of Hawkes process with the log-likelihood function given the actual occurrence pattern of a considered word observed in real written text. With that optimized Hawkes process, the occurrence signal of the word is generated and compared to the actual occurrence pattern of the word. The validity of the optimized Hawkes process was confirmed through comparisons between ACFs obtained from real word occurrence signals X(t) and those obtained from the simulated X(t). Degrees of agreement in various characteristic quantities of ACF show that Hawkes processes have a satisfactory ability to reproduce actual word occurrence signals in real written texts. Therefore, Hawkes processes can be utilized to express or simulate real word occurrence patterns of Type-I words. One of the advantages of using the Hawkes process in this manner is that it allows us to infer how a considered Type-I word works in a document. More specifically, we can determine the characters of the dynamic correlation of the word from the parameters vector, $\theta_{exp}$ or $\theta_{pow}$, although the accuracy of parameter estimation needs to be further improved for this purpose.

We further found that simulated word occurrence signals from Hawkes processes have a property that waiting time $t_{w}$ and its distribution $P\left(t_{w}\right)$ show linear relationship in double-logarithmic plots, indicating that the employed Hawkes processes have some fractal structure in their WTDs. The generalization of this finding through large scale simulations is an interesting theme for our future research.

Another possible direction of a future study is to establish a link between the stochastic model of Type-I words and some kind of diffusion model. Indeed, in our previous study [16], we utilized a Weiestrass random walk model [33,34,35] and modified it to derive the linear relationship between $\log t_{w}$ and $\log P\left(t_{w}\right)$ which was observed in WTDs of Type-I words. More generally, a methodology that directly relates point processes to diffusion processes has already been proposed [36,37]. This may allow us to apply various findings on fractional Brownian motion [38,39,40] to the analysis of the generation process of Type-I words.

This study confirms that the yielding processes of Type-I words in seven famous academic books can be described somewhat accurately by the Hawkes processes, which was established through the curve fittings in which ACFs of simulated signals X(t) generated from Hawkes processes are well fitted by the KWW function. This result leads to a new research question: can the ACFs of signals generated from Hawkes processes always be described by the KWW functions? Solving this problem requires either running large-scale simulations of a new design or deductive arguments by developing the relevant point process theory. This issue is also an interesting research direction for the future.

## Figures and Tables

**Table 1:** Summary of English texts employed.

Short Name	Title	Author	Vocabulary Size	Length in Sentences	Number of Type-I Words
Darwin	On the Origin of Species	Charles Darwin	5728	4036	124
Einstein	Relativity: The Special and General Theory	Albert Einstein	2222	1107	20
Freud	Dream Psychology	Sigmund Freud	4520	1977	18
Kant	The Critique of Pure Reason	Immanuel Kant	5157	5920	157
Lavoisier	Elements of Chemistry	Antoine Lavoisier	5558	3899	122
Plato	The Republic	Plato	5686	5268	49
Smith	An Inquiry into the Nature and Causes of the Wealth of Nations	Adam Smith	8399	11906	433

**Table 2:** Results of MLE of the Hawkes process with the word occurrence signal of “organ” in Darwin text.

Kernel Type	Parameters Vector	AIC	BIC
$g_{exp}\left(\tau \right)$	(μ,a,b)=(0.00893, 0.23944, 0.25068)	540.9253578	546.7790890
$g_{pow}\left(\tau \right)$	(μ,K,c,p)=(0.00743, 0.62804, 5.05592,1.56729)	541.3719225	549.1768974

**Table 3:** Fitting results for two ACFs of the actual and and the simulated signals.

Type of X(t)	Number of Word Occurrences	Fitting Parameters in Equation (3)			BIC
		α	β	τ	
observed signal in real written text	155	1.00000	0.23825	1.64135	−691.30057
simulated signal from Hawkes process	157	1.00000	0.27683	1.16607	−680.82002

## Data Availability

Not applicable.
